# Laparoscopic cholecystectomy in a patient with sickle cell disease and acquired cranial gallbladder malposition: technical adaptations to achieve safe dissection

**DOI:** 10.1093/jscr/rjag292

**Published:** 2026-04-23

**Authors:** Noor Albusta, Ammar Kheyami, Hamza Ahmed

**Affiliations:** Department of Internal Medicine, Lahey Hospital and Medical Center, 41 Mall Road, Burlington, MA 01805, United States; Department of General Surgery, Salmaniya Medical Complex, Road 2904, Block 329, Manama, Bahrain; Department of General Surgery, Salmaniya Medical Complex, Road 2904, Block 329, Manama, Bahrain

**Keywords:** hepatomegaly, cholecystectomy, sickle cell disease

## Abstract

We report the case of a 38-year-old male with sickle cell disease (SCD) who underwent elective laparoscopic cholecystectomy for recurrent biliary colic. Intraoperative exploration revealed a rare acquired cranial gallbladder malposition, with the gallbladder lying high in the subdiaphragmatic space and displaced posteriorly, in the setting of marked hepatomegaly and chronic inflammation. This abnormal positioning resulted in significant exposure limitations and difficulty achieving the classical critical view of safety. Conventional fundal traction was ineffective. Adequate exposure was ultimately achieved through direct retraction of the right hepatic lobe via the epigastric trocar, combined with surgeon repositioning and modified use of existing ports. The procedure was completed laparoscopically without conversion to open surgery or complications. This case highlights the importance of recognizing acquired gallbladder malposition as a cause of difficult cholecystectomy and underscores the need for adaptable operative strategies to achieve safe dissection.

## Introduction

Laparoscopic cholecystectomy is the standard treatment for symptomatic cholelithiasis and one of the most commonly performed abdominal operations worldwide [1]. Although generally safe, bile duct injury remains a serious complication, most often occurring in the setting of distorted anatomy or inadequate exposure [2]. Altered gallbladder position is a recognized cause of operative difficulty and increases the risk of misidentification during dissection [3].

True acquired malposition of the gallbladder is rare and has been described mainly in association with cirrhosis-related hepatic distortion [4, 5]. Patients with sickle cell disease (SCD) frequently develop pigment gallstones due to chronic hemolysis and often require cholecystectomy [6, 7]; however, the disease is not associated with congenital biliary anomalies [8]. Gallbladder malposition in these patients should therefore prompt consideration of an acquired process related to hepatomegaly and chronic hepatic remodeling.

We report a challenging laparoscopic cholecystectomy in a patient with SCD and marked hepatomegaly, in whom the gallbladder demonstrated an acquired cranial malposition with important implications for operative exposure and technique.

## Case presentation

A 38-year-old man with known glucose-6-phosphate dehydrogenase deficiency and SCD presented with intermittent colicky right upper quadrant pain consistent with symptomatic cholelithiasis. He had been clinically stable, with no vaso-occlusive crises for more than 5 years, and was maintained on folic acid alone. Preoperative evaluation revealed no contraindications to general anesthesia. Standard perioperative precautions for SCD, including adequate hydration and oxygenation, were implemented.

### Operative findings

A standard four-port laparoscopic approach was used. On entry into the peritoneal cavity, marked hepatomegaly was immediately apparent, with the liver occupying much of the upper abdomen. The gallbladder was identified high in the subdiaphragmatic space near the liver dome, with posterior displacement and a more vertical axis than the usual infero-anterior orientation.

The gallbladder itself was neither distended nor adherent to surrounding structures. However, conventional cephalad fundal traction failed to provide adequate exposure because of the cranial position of the gallbladder in combination with the heavy, enlarged liver. As a result, exposure was limited by increased depth, altered angulation, and restricted working space, although overall biliary anatomy appeared preserved.

### Technical adaptations and surgical procedure

**Figure 1:**
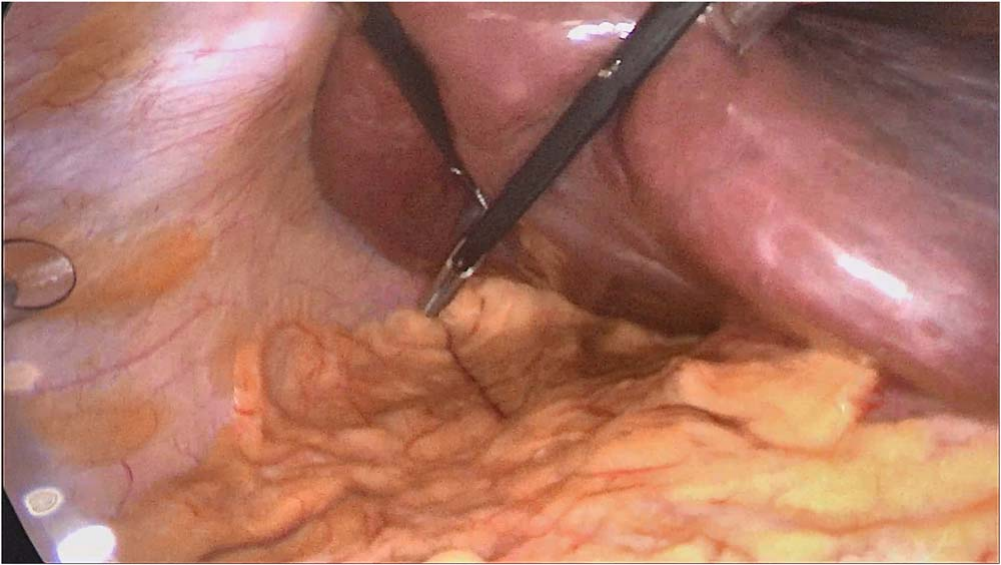
Intraoperative laparoscopic view demonstrating marked hepatomegaly with cranial, subdiaphragmatic displacement of the gallbladder. The gallbladder lies high under the liver dome with a distorted, more vertical axis, resulting in limited exposure of Calot’s triangle and failure of conventional fundal traction.

Given the failure of standard fundal traction, the operative strategy was modified. Direct retraction of the right hepatic lobe was performed using a grasper introduced through the epigastric trocar and applied to segments V and VI. Controlled anterior and caudal liver retraction was required to overcome the exposure limitations created by hepatomegaly and cranial gallbladder displacement (Fig. 1).

**Figure 2:**
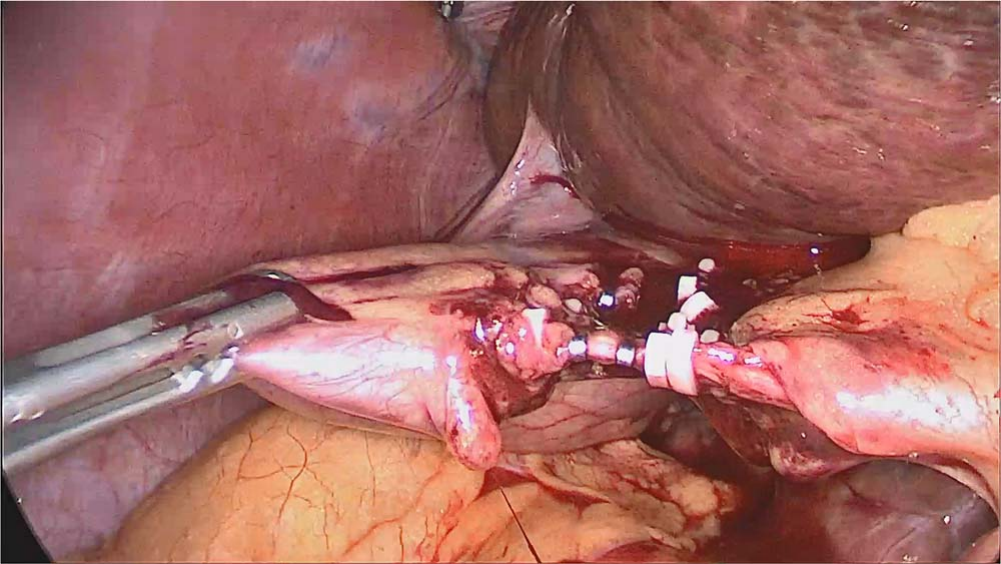
Modified exposure technique showing direct retraction of the right hepatic lobe to create an operative window. Liver retraction allowed visualization of Calot’s triangle and safe identification of the cystic duct and artery despite incomplete attainment of the classical critical view of safety.

To optimize ergonomics and instrument alignment, the primary surgeon repositioned to operate predominantly from the patient’s right side, utilizing the right lateral trocars. This adjustment, combined with direct liver retraction, compensated for the altered gallbladder axis and increased depth of the operative field, allowing visualization of Calot’s triangle (Fig. 2).

Meticulous dissection was undertaken, and the cystic duct and cystic artery were identified, clipped, and divided in a controlled manner. Dissection of the gallbladder from the liver bed was prolonged due to chronic inflammation and increased vascularity associated with hepatomegaly. The gallbladder was removed in an endoscopic retrieval bag through the epigastric port (Supplementary Video 1).

### Postoperative course

The patient had an uncomplicated recovery and was discharged with routine postoperative instructions. Histopathology confirmed chronic cholecystitis, consistent with intraoperative findings.

## Discussion

Gallbladder malposition is an uncommon but clinically relevant finding that can substantially increase the technical complexity of laparoscopic cholecystectomy. Congenital ectopic gallbladder positions have been well described, notably by Puente and Bannura, who classified biliary positional variants based on radiologic anatomy and embryologic migration patterns [8]. In contrast, true acquired gallbladder malposition remains poorly recognized in surgical practice.

When acquired malposition has been reported, it has most often been associated with cirrhosis and chronic liver disease. Hossack and Date demonstrated that hepatic architectural distortion can result in secondary displacement of the gallbladder and increased operative difficulty [4]. Hussain similarly emphasized that altered liver morphology can impair exposure and increase the risk of bile duct injury if standard laparoscopic techniques are applied without modification [5].

Anatomic and radiologic studies support the concept of secondary displacement rather than developmental abnormality. Chronic changes in liver volume and contour may alter spatial relationships of adjacent viscera without implying congenital anomaly. This distinction is important, as congenital ectopic gallbladders may be associated with anomalous biliary anatomy, whereas acquired malposition typically preserves ductal relationships but creates exposure-related challenges.

In the present case, the gallbladder demonstrated cranial, subdiaphragmatic displacement with posterior orientation in the setting of marked hepatomegaly. The absence of features suggestive of congenital biliary malformation, together with known hepatic manifestations of SCD, supports an acquired etiology. Chronic hemolysis predisposes to pigment gallstones and recurrent cholecystitis [6], while hepatic involvement in SCD—including congestion, vaso-occlusive injury, iron deposition, and extramedullary hematopoiesis—may contribute to hepatomegaly and hepatic remodeling [9, 10]. Over time, these processes may distort hepatic contours sufficiently to displace the gallbladder from its usual fossa, even without cirrhosis.

From a technical perspective, this case reinforces that difficult cholecystectomy in hepatomegaly is often a problem of exposure rather than dissection. Strasberg and colleagues demonstrated that bile duct injury most commonly results from misidentification under poor operative conditions rather than rare ductal anomalies [2, 11]. In this patient, conventional fundal traction failed to improve exposure, a recognized precursor to biliary injury.

Adoption of direct liver retraction combined with surgeon repositioning allowed safe progression without additional trocars or conversion. Such exposure-focused adaptations are advocated in difficult cholecystectomy [5, 12]. Although the critical view of safety remains central, it may not always be fully achievable in severe anatomic distortion, and contemporary guidelines emphasize prioritizing safety through exposure modification or bail-out strategies when necessary [13–15].

## Conclusion

This case highlights an uncommon cause of difficult laparoscopic cholecystectomy—acquired cranial gallbladder malposition due to hepatomegaly in SCD. Early recognition of exposure failure and operative adaptation enabled safe laparoscopic completion. Surgeons should consider acquired gallbladder displacement in patients with chronic liver enlargement and adopt flexible, anatomy-driven approaches.

## Supplementary Material

procedure_video_rjag292
